# Peroxiredoxin I maintains luteal function by regulating unfolded protein response

**DOI:** 10.1186/s12958-018-0396-0

**Published:** 2018-08-15

**Authors:** Hyo-Jin Park, Dong Gil Lee, Jung Bae Seong, Hyun-Shik Lee, Oh-Shin Kwon, Beom Sik Kang, Jeen-woo Park, Sang-Rae Lee, Dong-Seok Lee

**Affiliations:** 1 College of Engineering, Daegu University, Biotechnology, Gyeongsan, South Korea; 2 School of Life Sciences, BK21 Plus KNU Creative BioResearch Group, Kyungpook National University, Daegu, South Korea; 3 Korea Research Institute of Bioscience and Biotechnology (KRIBB), National Primate Research Center (NPRC), Daejeon, South Korea; 4 College of Natural Sciences, Kyungpook National University, Daegu, 702-701 Republic of Korea

**Keywords:** Corpus luteum, Peroxiredoxin 1, Unfolded protein response, Apoptosis

## Abstract

**Background:**

Mounting evidence shows that ROS regulation by various antioxidants is essential for the expression of enzymes involved in steroidogenesis and maintenance of progesterone production by the corpus luteum (CL). However, the underlying mechanisms of peroxiredoxin 1 (PRDX1), an antioxidant enzyme, in luteal function for progesterone production in mice have not been reported. The aim of this study was to evaluate the functional link between PRDX1 and progesterone production in the CL of *Prdx1* knockout (K/O) mice in the functional stage of CL.

**Methods:**

The expression pattern of the unfolded protein response (UPR) signaling pathways, endoplasmic reticulum (ER) stress-induced apoptosis related genes and peroxiredoxins 1 (PRDX1) were investigated by western blotting analysis in CL tissue of 10 weeks mice during functional stage of CL. The protein levels of these genes after ER-stress inducer tunicamycin (Tm), ER-stress inhibitor tauroursodeoxycholic acid (TUDCA) and ROS scavenger, N-acetylcysteine (NAC) stimulation by intraperitoneal (i.p) injection were also investigated in CL tissue of wild type (WT) mice. Finally, we examined progesterone production and UPR signaling related gene expression in CL tissue of *Prdx1* K/O mice.

**Results:**

We demonstrated that PRDX1 deficiency in the functional stage activates the UPR signaling pathways in response to ER stress-induced apoptosis. Interestingly, CL number, serum progesterone levels, and steroidogenic enzyme expression in *Prdx1* K/O mice decreased significantly, compared to those in wild type mice. Levels of UPR signaling pathway markers (GRP78/BIP, P50ATF6, and phosphorylated (p)-eIF2) and ER-stress associated apoptotic factors (CHOP, p-JNK, and cleaved caspase-3) were dramatically increased in the CL tissue of *Prdx1* K/O mice. In addition, administration of the NAC, reduced progesterone production and activated ER-stress-induced UPR signaling in the CL tissue obtained from the ovary of *Prdx1* K/O mice. Taken together, these results indicated that reduction in serum progesterone levels and activation of ER-stress-induced UPR signaling are restored by NAC injection in the CL of *Prdx1* K/O mice.

**Conclusion:**

These observations provide the first evidence regarding the basic mechanisms connecting PRDX1 and progesterone production in the functional stage of CL.

**Electronic supplementary material:**

The online version of this article (10.1186/s12958-018-0396-0) contains supplementary material, which is available to authorized users.

## Background

Regulation of antioxidant enzymes and reactive oxygen species (ROS) production has been proposed as a major mediator of granulosa/cumulus cell communication, oocyte maturation [1], and luteal phase development in mice [2]. Previous studies showed that abnormal expression of antioxidant enzymes is associated with ROS production in female reproductive system such as those affecting adverse pregnancy outcomes [3], resumption of meiosis, ovarian functions, and reducing fertility [4, 5]. In particular, excess ROS production, especially in the corpus luteum (CL), induces hormone imbalance and luteal phase defect in female mice [5]. To protect cells against the damaging and toxic effects of ROS, antioxidant enzymes, including catalase (CAT), glutathione peroxidase (GPX), superoxide dismutase (SOD), and peroxiredoxins (PRDXs) are activated in mammalian cells [6, 7]. The peroxiredoxin family consists of six proteins (PRDX1–6), which can reduce hydrogen peroxide (H_2_O_2_), lipid hydroperoxides, and peroxynitrite. PRDX 1–6 are categorized as typical 2-Cys peroxiredoxins, which are distinct from the atypical 2-Cys (PRDX5) and 1-Cys peroxiredoxins (PRDX 6) [8]. Among these PRDXs, PRDX1 is localized in the cytosol, where it interacts with various signaling molecules [9, 10]. In addition, a relationship between granulosa cell stimulation and PRDX1 expression has been previously detected in the rat ovary [10].

The CL is a transient ovarian endocrine gland, which secretes progesterone, a hormone essential for regulation of the luteal phase [11]. Steroidogenic enzymes such as steroidogenic acute regulatory (StAR) protein, p450 cholesterol side-chain cleavage enzyme (P450scc), and 3β-hydroxysteroid dehydrogenase (3β-HSD) are required [12, 13] for progesterone production by the luteal cells of the CL. Loss of progesterone production, followed by loss of luteal cell function, leads to CL regression [11]. Recent studies showed that H_2_O_2_ production from the CL tissue is important for luteal function, such as for steroidogenic enzyme activation and regression of CL during the luteal phase [14]. Therefore, regulation of ROS production can play an important role in progesterone production and maintenance of luteal functions during the CL life span. However, the mechanisms via which PRDX1 acts as a ROS regulator and enables progesterone production in mice CL have not been reported.

ROS production is induced by endoplasmic reticulum (ER) stress and activation of unfolded protein response (UPR) [15]. The UPR alleviates ER stress, rescues ER homeostasis, and prevents cell death via the induction of ER chaperone expression, reduction of protein synthesis, and degradation of unfolded and/or misfolded proteins using three ER-localized transmembrane proteins, namely, inositol-requiring enzyme 1 (IRE1 or ERN1), protein kinase RNA (PKR)-like ER kinase (PERK), and activating transcription factor-6 (ATF6) [16]. Therefore, all three UPR pathways contribute to the induction of cell apoptosis or ER stress-associated cell death, under conditions of excessive ER stress. In addition, it is well known that C/EBP homologous protein (CHOP), cJUN NH2-terminal kinase (JNK) and caspase-12 have been implicated in apoptotic signaling in response to ER stress [17]. Previously, we demonstrated that ER stress affects progesterone production during the functional stage of CL in mice and bovine CL tissue of estrous cycle [18, 19]. Additionally, reports show that PRDX4 protects against ER-stress mediated cell death by removing H_2_O_2_ [20]. However, the roles of PRDX1 in regulation of ER stress and UPR signaling pathways during progesterone production in the functional stage of CL has not yet been investigated.

Therefore, the aims of present study were: i) to investigate whether PRDX1 acts as an antioxidant and ROS-regulator during progesterone production, ii) to confirm the association of PRDX1 with oxidative stress-derived ER stress response and UPR activation using an ER stress inducer and inhibitor during the functional stage of CL in mice, and iii) to evaluate the changes in progesterone production, UPR signaling, and ER stress-mediated apoptosis in the CL tissue of *Prdx1* knockout (K/O) mice compared to those in wild type (WT) mice. Finally, we investigated whether injection of the ROS scavenger, N-acetylcysteine (NAC), in *Prdx1* K/O mice effectively controls progesterone production by altering the levels of enzymes involved in steroidogenesis and regulating ER stress in mice CL.

## Methods

### Chemicals

Unless otherwise stated, all chemicals and reagents used in this study were purchased from Sigma Aldrich (St. Louis, MO, USA).

### Animals

Female C57/B6J mice (8–10-week-old) of wild type were purchased from Hyochang Bio-Science (Daegu, Korea) and *Prdx1*-deficient (*Prdx1* −/−) female mice (8–10-weeks old) maintained in accordance with the institutional guidelines of the Institutional Animal Care and Use Committee of the Korea Research Institute of Bioscience and Biotechnology (KRIBB, Daejeon, South Korea). Animals were maintained under standard environmental conditions (temperature at 20–22 °C, humidity at 50–60%, and 12-h-dark/light cycles) with free access to food and water.

### Determination of estrous cycle

**Fig. 1:**
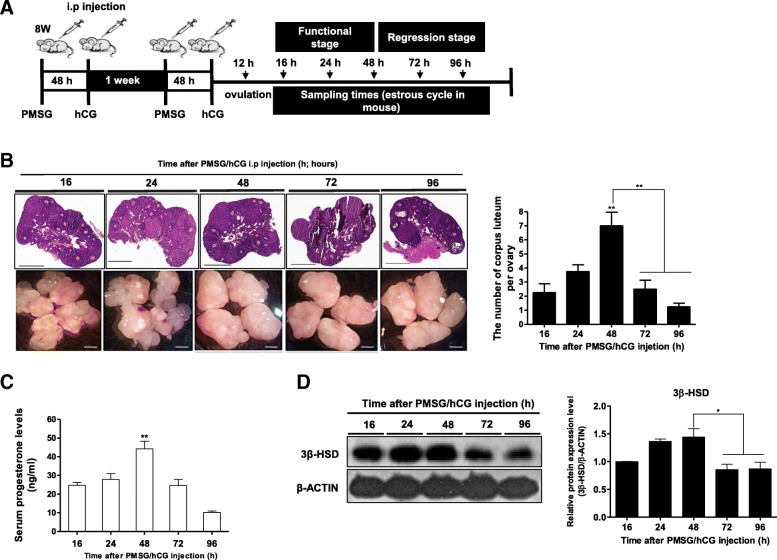
The number of CL, serum progesterone and 3β-HSD expression were increased 48 h to peak levels of progesterone production after PMSG/hCG injection. **a** Schematic diagram of ovary tissue selected 16, 24, 48, 72, and 96 h after PMSG/hCG injection. CL tissues were carefully collected from the ovaries after ovulation. **b** Luteal phase samples were collected at different time points based on CL morphology. **c** Serum progesterone levels were determined by progesterone ELISA kit (ALPCO) for 4–5 animals at each time point of the luteal phase during the estrous cycle. **d** Western blotting analysis of 3β-HSD, a marker of progesterone synthesis in the CL. The 3β-HSD level was normalized to that of the β-actin control. The histogram represents values of densitometry analysis obtained using the Image J software (NIH). The data are representative of at least three independent experiments conducted in triplicate. Values represent means ± SEM (4–5 mice/group) * *P* < 0.05, **P < 0.01, and ***P < 0.001

The short reproductive cycle length observed in rodents is called the estrous cycle. The normal estrous cycle in rodents follows a 4–5-day pattern, the characteristics of which vary with the day [21]. We classified mice on the basis of the predicted time of hormone induced-superovulation as described previously [18]. In all mice, superovulation was achieved by intraperitoneal (i.p) injection of 5 IU pregnant mare’s serum gonadotropin/mouse (*n* = 5–6 in each group, 8-week-old) (PMSG; Sigma Aldrich), followed 48 h later by injection of 5 IU human chorionic gonadotropin/mouse (hCG; Sigma Aldrich). Ovulation occurred 12 h after the hCG injection, and therefore, 16 h post-hCG injection was considered as the beginning of CL formation. CL tissues were collected from the ovaries of treated mice 16, 24, 48, 72 and 96 h after hCG administration (Fig. 1a). The luteal phase was divided into two groups, namely, the functional stage (16, 24 and 48 h) and the regressing stage (72, 96 h) on the basis of serum progesterone concentration and changes in steroidogenic enzyme expression during progesterone production. Mice were killed, blood was collected, and the ovaries were removed at the indicated time intervals. CL tissues were collected from the ovaries under a dissecting microscope and immediately frozen at − 70 °C until further use. All experiments were performed in triplicate, and each experiment was independently analyzed.

### Administration of tm, TUDCA, and NAC in female mice

C57BL/6 female mice (8-week-old) were administered tunicamycin (Tm; 0.5 μg/g body weight; Calbiochem, CA, USA) as an ER stress inducer, and tauroursodeoxycholic acid (TUDCA;0.5 μg/g body weight; Calbiochem) or NAC (1.0 μg/g, Sigma) as ER stress inhibitors by i.p. injection at the indicated times (16 h). Saline was injected intraperitoneally in the control animals. After administration, the mice were sacrificed at the indicated times, and blood and CL tissues were collected.

### Progesterone estimation by enzyme immunoassay (EIA)

For estimating serum progesterone concentration, blood was collected from female mice after each injection (16, 24, 48, 72 and 96 h after PMSG/hCG treatment). Serum was separated from blood and stored at − 20 °C until it was assayed for progesterone. Progesterone concentration was assessed using a progesterone EIA kit (ALPCO, Windham, NH, USA) according to the manufacturer’s instructions.

### RNA extraction and reverse transcription polymerase chain reaction (RT-PCR)

Total RNA was isolated from each individual CL tissue using the Trizol reagent (Invitrogen**,** Inc., CA, USA) according to the manufacturer’s instructions. RNA concentration and purity were measured using a NanoDrop spectrophotometer (ASP-2680; ACTgene, NJ, USA). Next, 1 μg/μl total RNA and the AccuPower® RT-PCR premix (Bioneer Inc., Daejeon, South Korea) were used to synthesize the cDNA. Primers specific for the mouse sequences of interest (Table 1) were designed using the National Center for Biotechnology Information (NCBI) database. PCR was performed using the following condition: 95 °C for 5 min, 30 cycles of 95 °C for 30 s, 55~ 60 °C for 30 s, 72 °C for 30 s, and 72 °C for 5 min. The PCR products were separated by electrophoresis on a 2% agarose gel with a known standard (100 bp ladder, Bioneer Inc.), stained with ethidium bromide, and photographed under UV illumination. Band intensities were quantified using the Image J software (National Institute of Health, Bethesda, MD, USA).

**Table 1:** Primer sequence for reverse transcription PCR

Target	Accession numbers	Primer	Sequence reported 5′-3′	Tm°C	length (bp)
3β-hsd	NM_008293.3	Forward:5’	ACTGCAGGAGGTCAGAGCT	55	401
		Reverse:3’	GCCAGTAACACACAGAATACC		
Prdx1	NM_011034.4	Forward:5’	CACCCAAGAAACAAGGAGGA	53.5	343
		Reverse:3’	TGGTCCAGTGCTCACTTCTG		
Star	NM_011485.4	Forward:5’	GAAAAGACACGGTCATCACT	56	262
		Reverse:3’	CCGTGTCTTTTCCAATCCTC		
P450scc (Cyp11a1)	AF195119.1	Forward:5’	GCTGGAAGGTGTAGCTCAGG	57	224
		Reverse:3’	CACTGGTGTGGAACATCTGG		
Gapdh	BC145810.1	Forward:5’	ACCACAGTCCATGCCATCAC	55	452
		Reverse:3’	TCCACCACCCTGTTGCTGTA		

*3β-hsd*: 3β-hydroxysteroid dehydrogenase, *Prdx1*: peroxiredoxin 1, *Star*: steroidogenesis acute regulatory, *P450scc*: cytochrome P450 side-chain cleavage, Tm: melting temperature

### Protein extraction and western blotting

Lysates of CL tissue from each stage were prepared in PRO-PREP protein lysis buffer (iNtRON, Daejeon, Korea). The protein concentration for each sample was estimated using a Bradford dye-binding assay with bovine serum albumin (BSA) as a standard (Bradford, 1976). Aliquots of the proteins (30 μg) were separated by sodium dodecyl sulfate-polyacrylamide gel electrophoresis (SDS-PAGE) on 12% gels. After electrophoresis, the separated proteins were transferred onto nitrocellulose (NC) membranes (Pall Corporation, Port Washington, NY, USA). After blocking with 5% non-fat milk in Tris-buffered saline (TBS) with 0.1% Tween 20 at 4 °C under mild agitation, the membranes were incubated with 1:2000 diluted anti-CHOP, anti-p90ATF6, anti-CREB-2, anti-3β-HSD (Santa Cruz, Biotechnology, CA, USA), anti-GRP78/BIP, anti-p-eIF2α, anti-IRE1, anti-eIF2a, anti-JNK, anti-p-SAPK/JNK, anti-caspase-3, anti- β-tubulin (Cell Signaling, Beverly, MA, USA), anti-p50ATF6 (IMG, San Diego, CA, USA), and anti-p-IRE1α (Abcam, Cambridge, MA, USA) antibodies. The membranes were then incubated with secondary horse radish peroxidase (HRP)-conjugated anti-goat/mouse/rabbit IgG (Thermo, Rockford, IL). Antibody binding was detected using an enhanced chemiluminescence (ECL) kit (Advansta, CA, USA). Band intensities were quantified using the Image J software (NIH, MD, USA).

### Hematoxylin & eosin staining

The ovaries were isolated from the body and fixed with 4% formalin (Sigma Aldrich). The sections were stained with hematoxylin and eosin using a standard protocol described previously (Park, et al. 2013b, Park, et al. 2014) [18, 22]. Thin sections were cut with a diamond knife and mounted. The sections were observed under an Olympus BX51 microscope (Olympus, Tokyo, Japan).

### Immunohistochemistry

The ovaries were fixed in formalin, embedded in paraffin, and cut into 3-μm-thick sections. The sections were deparaffinized and briefly heated. The sections were then treated with a protein blocking solution (Dako, CA, USA) and incubated with anti-phospho eIF2a (Cell Signaling). After washing with 0.1 M TBS containing 0.01% Tween-20 (TBST), the sections were incubated with anti-rabbit polymer (Dako). Peroxidases bound to the antibody complex were visualized after treatment with a 3, 3′-diaminobenzidine (DAB) chromogen substrate solution (Dako). The DAB reaction was monitored under a microscope to determine the optimal incubation time and stopped with several washes of 0.1 M TBS. The immunolabeled sections were dehydrated in a graded ethanol series, defatted in xylene, and mounted. The sections were examined under a bright field Olympus BX51 microscope and images were acquired using an Olympus DP 70 camera (Olympus, Japan).

### Statistical analysis

All experiments were repeated at least three times, and percentage data obtained in this study are presented as the mean ± standard error of the mean (SEM). These data were subjected to one-way analysis of variance (ANOVA), followed by Dunnett’s multiple comparison test. Statistical analysis was performed using paired Student’s t-test. Data obtained from WT and *Prdx1* K/O mice CL tissues were compared using the unpaired Student’s t-test. All calculations were performed using the GraphPad Prism 5.0 software package (GraphPad Software, San Diego, CA, USA). *P* < 0.05 was considered to be statistically significant.

## Results

### Measurement of progesterone production in CL tissue of mouse

For delineating the timing of the luteal phase during the estrous cycle post-PMSG/hCG injection, we used a methods of hormone injection for synchronization of estrous cycle as previously described by Park et al., [18].

We observed the morphology and determined the number of collected CL tissues microscopically at 16, 24, 48, 72 and 96 h after PMSG/hCG injection (Fig. 1a and b). Next, we determined the serum progesterone level and the expression of 3β-HSD, a classical steroidogenic enzyme involved in progesterone biosynthesis, using the progesterone ELISA kit (ALPCO) and western blot analysis during mice estrous cycle, respectively. Serum progesterone level was significantly increased (*P* < 0.01) 48 h after the PMSG/hCG injection (Fig. 1c). Protein levels of 3β-HSD showed the same pattern as that of serum progesterone till the CL functional stage, but rapidly decreased after 48 h (Fig. 1d). Based on these results, we concluded that the time point (48 h) corresponding to peak progesterone levels in CL tissues during the entire luteal phase after PMSG/hCG injection should be used for subsequent experiments.

### ER-stress-induced reduction in progesterone production at the functional stage of CL is recovered by the ROS scavenger NAC

**Fig. 2:**
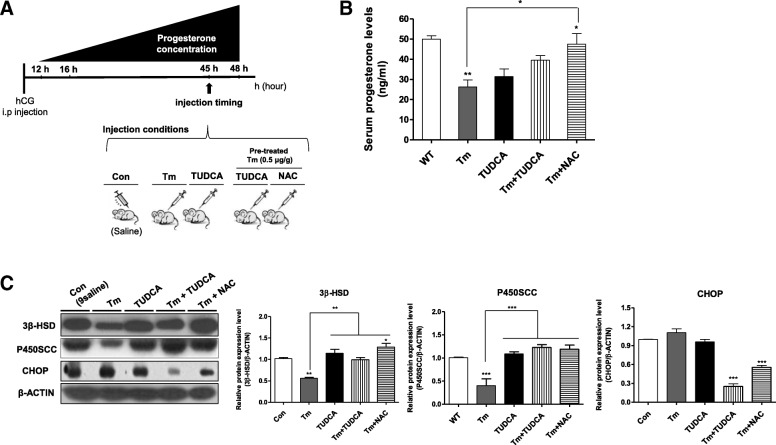
ER-stress-induced reduction in progesterone production at the functional stage of CL is recovered by ROS scavenger NAC. **a** Schematic diagram showing the injection and collection times of the ER stress-regulator and ROS scavenger injection. Tm (an ER stress inducer; 0.5 μg/g body weight), TUDCA, (an ER stress inhibitor; 0.5 μg/g body weight), or NAC (a ROS scavenger; 1.0 μg/g body weight) were injected into mice 45 h after the PMSG/hCG injection. **b** Peak production of serum progesterone was measured (48 h after the PMSG/hCG injection) in the luteal phase. **c** Protein level of the UPR signaling activation marker CHOP in selected CL tissues was measured by western blot analysis after ER stress regulator and ROS scavenger stimulation. The levels of 3β-HSD, P450scc, and CHOP were normalized to that of β-actin. The histogram represents values of densitometry analysis obtained using the Image J software (NIH). The bar graphs represent the least-squares means ± SEM (4–5 mice/group) of three independent experiments. * *P* < 0.05, ** *P* < 0.01, and *** *P* < 0.001; Dunnett’s multiple comparison test compared to 48 h after PMSG/hCG injection

In our previous study, we demonstrated that ER stress affects progesterone production during the functional stage of CL. In addition, ER stress-mediated apoptosis induced CL regression in the mouse luteal phase [18]. Recent studies indicate that ROS production can be induced by ER stress [23]. Therefore, we investigated whether progesterone production is regulated by the ROS scavenger under ER stress in the functional stage of CL. To analyze the relationship between ROS and ER stress during progesterone production in the CL tissue at the peak of progesterone synthesis (48 h after PMSG/hCG injection), we performed the experiment as shown in Fig. 2a. We used Tm as an ER stress inducer, TUDCA as an ER stress inhibitor, or NAC as a ROS scavenger. Mice injected with Tm (0.5 μg/g) showed dynamic deficiency of luteal function with low serum progesterone and reduction in 3β-HSD and P450scc protein levels in the CL. Compared to the Tm-treated group, serum progesterone level recovered significantly (*P* < 0.05) in the group where NAC (1.0 μg/g) was added after Tm (0.5 μg/g) pre-treatment (Fig. 2b). Addition of NAC as a recovery effect like a TUDCA also significantly increased the protein levels of 3β-HSD and P450scc in CL (Fig. 2c). On the contrary, NAC or TUDCA administration in mice pre-treated with Tm decreased the expression level of CHOP as an ER stress-mediated apoptotic factors during the functional stage of CL (*P* < 0.001) (Fig. 2c). These results demonstrate that the regulation of ER stress-induced ROS production is required for maintaining progesterone production during the functional stage of CL in mice.

### Prdx1 knockout reduced CL number, and levels of serum progesterone, 3β-HSD, and P450ssc

The above results underline the necessity of regulating ROS synthesis for maintaining progesterone production during the functional stage of CL (Fig. 2). We investigated whether PRDXs can act as antioxidants for maintaining progesterone production.

**Fig. 3:**
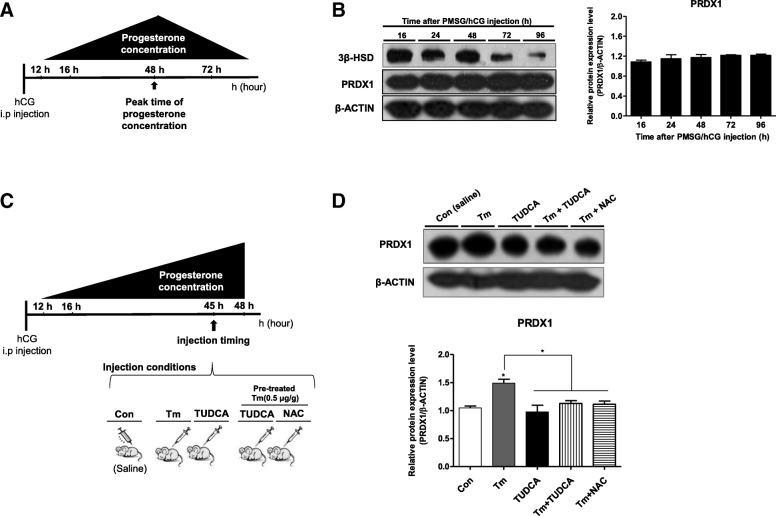
Increased PRDX1 expression by injection of ER-stress inducer (Tm) in the CL tissue was recovered by ER-stress inhibitor (TUDCA) or ROS scavenger (NAC). **a** Schematic diagram showing the time taken to reach peak progesterone levels in CL 48 h after PMSG/hCG injection. **b** Protein levels of PRDX1 and 3β-HSD were measured by western blot analysis in different sampling times of the luteal phase. **c** Schematic diagram showing the timing of ER stress-regulator and ROS scavenger injection and sample collection. **d** PRDX1 levels in selected CL tissues were measured using western blot analysis in the presence of the ER stress inducer (Tm) (0.5 μg/g body weight) or ER stress inhibitor (TUDCA) (0.5 μg/g body weight). And ER stress inhibitor (TUDCA) (0.5 μg/g body weight) or ROS scavenger (NAC) (1.0 μg/g body weight) after pre-treatment with the ER stress inducer (0.5 μg/g Tm), respectively. 3β-HSD and PRDX1 levels were normalized to that of β-actin. The histogram represents values densitometry analysis was performed using the Image J software (NIH). The bar graphs represent the least-squares means ± SEM (4–5 mice/group) of three independent experiments. * *P* < 0.05, **P < 0.01, and ***P < 0.001

**Fig. 4:**
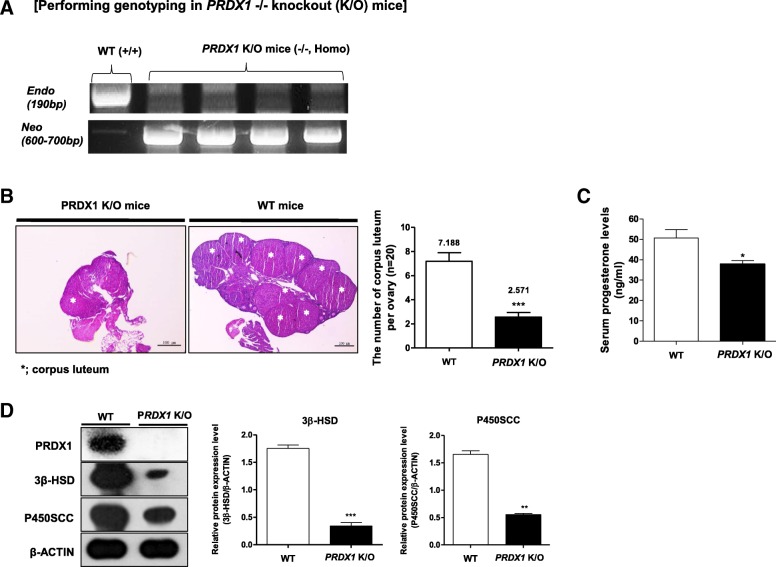
The CL number, serum progesterone, 3β-HSD, and P450ssc expression were decreased in *Prdx1* K/O mice. **a** We performed genotyping of PRDX1 by PCR in the tail of *Prdx1* K/O mice. **b** CL number in the ovary of *Prdx1* K/O mice was observed by hematoxylin and eosin (H & E) staining. **c** Serum progesterone levels were measured using a progesterone kit in *Prdx1* K/O mice. **d** The levels of the steroidogenic enzymes 3β-HSD and P450SCC were determined in CL tissue by western blot analysis. The relative levels of these proteins were obtained after normalization with β-actin levels. The histogram values of densitometry analysis were obtained using the Image J software. The bars represent the mean of two independent experiments means ± SEM (4–5 mice/group) * *P* < 0.05, **P < 0.01, and ***P < 0.001; two-tailed Student’s *t*-test compared to WT mice

The expression of the PRDXs at 16, 24, 48, 72 and 96 h after PMSG/hCG injection were analyzed (Fig. 3a). Interestingly, PRDX1 was continuously expressed in CL during the entire luteal phase (Fig. 3b), irrespective of the changes in 3β-HSD expression. In contrast, western blotting showed that the other PRDX proteins (2–6) were not expressed continuously during the entire luteal phase (Additional file 1: Figure S1). The PRDX1 level was increased by Tm treatment and recovered by TUDCA at the peak time point of progesterone production (Fig. 3c and d). TUDCA or NAC injection after Tm also decreased PRDX1 level compared to Tm only treatment (Fig. 3c and d). These results indicated that continuously expressed PRDX1 in CL during the entire luteal phase may be involved in ROS regulation for maintaining progesterone production in the functional stage of CL. Therefore, to investigate whether PRDX1 acts as an antioxidant and ROS-regulator during progesterone production in the functional luteal stage after PMSG/hCG injection, we evaluated the changes in progesterone production and expression of ER stress marker genes in the CL tissue of *Prdx1* K/O mice and compared them with those of WT mice. First, to determine the CL number, the ovary from *Prdx1* K/O and WT mice were subjected to genotyping and H & E staining, respectively (Fig. 4a). The number of formed CL and ovary size in *Prdx1* K/O mice dramatically decreased (*P* < 0.01; fold 2.8, Fig. 4b) 48 h after the PMSG/hCG injection. Similarly, the serum progesterone concentration and protein levels of 3β-HSD and P450ssc were significantly reduced compared to those in the CL of WT mice (Fig. 4c and d). Furthermore, to determine the effect of PRDX1 on progesterone production in CL tissue, we observed the ovary morphology in wild type, and *Prdx1* heterozygous and homozygous K/O mice. Although the ovary size in *Prdx1* heterozygous mouse was slightly reduced, the levels of serum progesterone and steroidogenic enzymes in the CL tissue of *Prdx1* heterozygous mice showed the same pattern as that of WT mice 48 h after PMSG/hCG injection (Additional file 2: Figure S2). These results demonstrate that absence of PRDX1 in the functional luteal stage after PMSG/hCG injection decreased CL number and ovary size compared to those of WT mice, which reduces serum progesterone and protein levels of the steroidogenesis enzymes 3β-HSD and P450ssc.

### PRDX1 deficiency induced UPR signal activation and ER stress-mediated apoptosis in CL functional stage

**Fig. 5:**
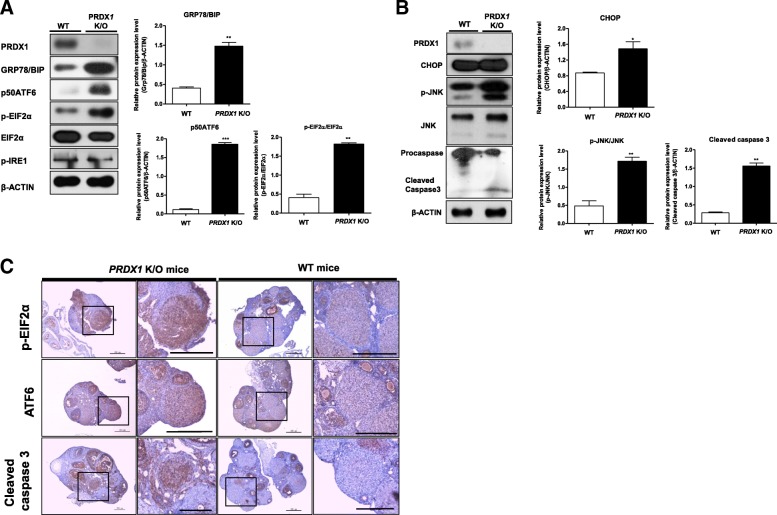
Protein levels of UPR signal activation and ER stress-mediated apoptosis in CL functional stage were induced in the CL tissue of *Prdx1* K/O mice. **a** Western blot analysis to evaluate the levels of PRDX1 and UPR signaling-related proteins (GRP78/BIP, p-EIF2α, EIF2α, p50ATF5, and p-IRE1) and (**b**) ER stress-mediated apoptotic factors (CHOP, p-JNK, JNK, and caspase-3) in the CL. The relative levels of these proteins were obtained after normalization with β-actin levels. The histogram values of densitometry analysis were obtained using the Image J software. The bars represent the means of two independent experiments ± SEM (4–5 mice/per group) * *P* < 0.05, **P < 0.01, and ***P < 0.001; two-tailed Student’s t-test compared to WT mice. **c** IHC staining for UPR markers (p-EIF2α and ATF6) and ER stress apoptosis marker (cleaved caspase-3) in CL tissue from ovary of *Prdx1* K/O mice. Scale bar = 200 μm

We investigated whether PRDX1 deficiency is involved in UPR signaling (GRP78/BIP, p-EIF2α, p50ATF6, and p-IRE1) or ER stress-mediated apoptosis (CHOP, p-JNK, and cleaved caspase-3) in the functional luteal stage using western blotting analysis and the CL tissue of WT and *Prdx1* K/O mice. As shown in Fig. 5a, the levels of GRP78/BIP, p50ATF6, and p-EIF2α were dramatically increased (*P* < 0.01) in the CL tissue of *Prdx1* K/O mice, but, the levels of total EIF2α and p-IRE1 showed no change compared to those of WT mice. The levels of CHOP, p-JNK, and cleaved caspase-3 were also significantly increased (CHOP; *P* < 0.05, p-JNK, and cleaved caspase-3; *P* < 0.01) in the CL of *Prdx1* K/O mice (Fig. 5b). In addition, immunohistochemistry (IHC) showed that the levels of p-EIF2α, ATF6, and cleaved caspase-3 were higher in the ovary of *Prdx1* K/O mice than in the ovary of WT mice (Fig. 5c). Therefore, these results demonstrated that PRDX1 deficiency during functional luteal stage activates the UPR signaling pathways in response to ER stress-mediated apoptosis.

### Restoration of impaired progesterone production and UPR signaling activation in response to ER stress by NAC injection in Prdx1 knockout mice

**Fig. 6:**
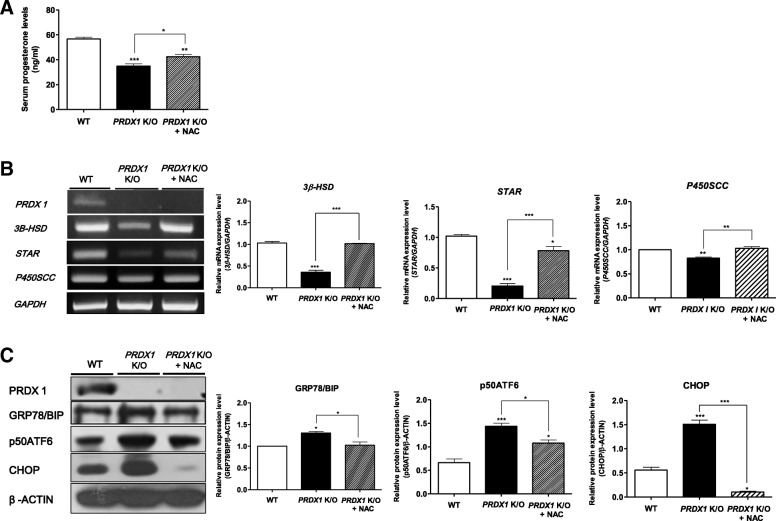
Deceased serum progesterone, steroidogenesis enzymes, and increased activation of UPR signaling in response to ER stress in *Prdx1* K/O mice are restored by injecting NAC, a ROS scavenger. **a** Serum progesterone levels of PRDX1 were measured using a progesterone kit after NAC (1.0 μg/g) i.p injection. **b** RT-PCR analysis of *Prdx1* and three major genes encoding steroidogenesis enzymes (3β-HSD*,* StAR, and P450scc). **c** The levels of PRDX1, UPR signaling-related proteins (GRP78/BIP and p50ATF5), and ER stress-mediated apoptotic factors (CHOP) were determined in the CL tissue of *Prdx1* K/O mice by western blot analysis after NAC (1.0 μg/g) i.p injection. The relative mRNA/protein levels were normalized to those of GAPDH/β-actin controls. The histogram values of densitometry analysis were obtained using Image J software. The data are representative of at least three independent experiments conducted in triplicate and shown as means ± SEM (3–5 mice/group). * *P* < 0.05, **P < 0.01, and ***P < 0.001; Dunnett’s multiple comparison test, compared to 48 h after PMSG/hCG injection

As shown in Fig. 2, the NAC-mediated decrease in ER stress-induced ROS levels is required for maintaining progesterone production during the luteal functional stage. Thus, we investigated whether NAC injection in *Prdx1* K/O mice restores the reduced serum progesterone concentration and protein levels of steroidogenesis enzymes in the ovaries of *Prdx1* K/O mice. First, we injected NAC 45 h after the PMSG/hCG injection, and collected CL tissue from the ovary 3 h after the NAC injection. As shown in the Fig. 6a, serum progesterone production, which was rapidly reduced (*P* < 0.01; compared to WT mice) in the CL tissue of *Prdx1* K/O mice, recovered significantly (*P* < 0.05; compared to *Prdx1* K/O mice) after the NAC injection. We also investigated whether NAC treatment of *Prdx1* K/O mice recovered steroidogenic enzyme expression and decreased activation of UPR signaling in response to ER stress during the functional luteal stage. We observed that the mRNA levels of the steroidogenic enzymes 3β-HSD, StAR (*P* < 0.001; compared to *Prdx1* K/O mice), and P450scc (*P* < 0.01; compared to *Prdx1* K/O mice) were recovered by NAC treatment (Fig. 6b). In contrast, the mRNA levels of CHOP (*P* < 0.001 compared to *Prdx1* K/O mice), GRP78/BIP, and p50ATF6 (*P* < 0.05 compared to *Prdx1* K/O mice) in the CL tissue of NAC-treated *Prdx1* K/O mice were significantly reduced (Fig. 6c). These results suggested that decrease in serum progesterone production and activation of UPR signaling in response to ER stress are restored by injecting NAC, a ROS scavenger, during the luteal stage of PRDX I KO mice.

## Discussion

In this study, we demonstrated that the antioxidant activity of PRDX1 is required for maintaining progesterone production and the protein levels of enzymes involved in steroidogenesis via regulation of the UPR signaling pathway and ER stress response in the functional stage of mice CL. In addition, injection of the ROS scavenger NAC restored the reduction in progesterone production and activation of the UPR signaling pathway in response to ER stress in the CL tissue of *Prdx1* K/O mice. Therefore, PRDX1 may act as a regulator for progesterone production by reducing ER stress during the luteal functional stage.

It is well known that regulation of ROS production by various antioxidant enzymes is essential for the expression of enzymes related to steroidogenesis, and maintenance of progesterone production and hormone secretion by the CL [24]. During the secretory phase of the CL, H_2_O_2_ is produced from the CL tissue by monooxygenation, which is catalyzed by the P450scc steroidogenic enzyme [14, 25]. The differential expression of antioxidant enzymes such as superoxide dismutase (SOD), catalase, and peroxiredoxins (PRDXs) suggest that oxidative stress affects ROS-related cellular mechanisms and the steroidogenic status of rat CL [26] and the bovine estrous cycle [27]. In this respect, although the roles of various antioxidant enzymes have been reported for female reproduction [5, 28], no direct information is available regarding the PRDX1 antioxidant system and its effect on progesterone production during the luteal phase.

Among these PRDXs, the protein levels of PRDX1 were continuously and highly expressed in the CL during the entire luteal phase (Fig. 3b). The levels of PRDX1, which protects against ER stress-mediated cell death by removing H_2_O_2_, was increased in the CL regression stage (Additional file 1: Figure S1). Protein level patterns of PRDX2, 3, 5, and 6 were irregularly expressed unrelated to CL formation, maintenance and regression progression in the CL during the entire luteal phase. The continuous high production of PRDX1 may stimulate ROS production from the CL tissue due to active metabolism and steroidogenesis during entire luteal phase (16–96 h PMSG/hCG injection). In addition, in *Prdx1* K/O mice, the number of CL tissues in the ovary, serum progesterone levels, and expression of the main steroidogenesis enzymes were dramatically reduced compared to those in WT mice (Fig. 4). The luteal phase requires adequate secretions of progesterone by the CL [29]. In luteal phase deficiency or CL insufficiency is widely accepted to be female reproductive function defects, perhaps due to poor follicular development and an abnormality of ovarian function [30, 31]. Also, luteal phase defect is induced an ovulatory disorder of considerable clinical importance that is implicated in infertility and recurrent spontaneous abortion [32]. As a results of Fig. 4, depending on the decrease in the size of the ovaries in Prdx1 K/O mice, we speculated that the CL formation number and progesterone production level at CL functional stage (48 h) can reduced. Also, we analogized that it would appear that impaired follicular growth and ovulation can occur by decreased in CL formation number in Prdx1 K/O mice. Therefore, our results demonstrate a link between the antioxidant enzyme PRDX1 and progesterone production in the CL of *Prdx1* K/O mice, which may be critical in regulating ROS production by the anti-oxidative effects of PRDX1 during the functional stage of mouse CL.

The ER stress-associated oxidative environment may also be correlated with ER stress-associated ROS production [33]. ROS is generated during protein unfolding due to the depletion of antioxidant enzymes [33]. Thus, secreted proteins such as steroid hormones involved in ER-regulated protein folding may be more susceptible to higher levels of oxidative stress. ER stress and oxidative stress are closely linked events [33]. Reports show that prolonged ER stress leads to ROS production and oxidative stress via ER stress-mediated pro-apoptotic signaling [34, 35]. We believe that the ER of luteal cells plays an important role as the site for steroidogenic enzyme synthesis and progesterone production during the CL functional stage. Previously, we observed that the progesterone production capacity of the CL tissue reduced after ER-stress inducer treatment [18].

We investigated whether N-acetyl-L-cysteine (NAC), which is widely used as a thiol-containing antioxidant [36], could protect progesterone production in WT mice after Tm administration. We also verified whether reduced progesterone production of *Prdx1* K/O mice could be rescued by NAC administration. As shown in Fig. 2, serum progesterone level was recovered by NAC treatment in Tm pre-treated WT mice. In addition, the levels of 3β-HSD and P450scc steroidogenic enzymes were recovered and that of CHOP (ER stress-mediated apoptotic factor) was reduced by NAC or TUDCA treatment. Based on these results, we concluded that the regulation of ER stress-induced ROS is required for maintaining progesterone production during the mouse luteal phase. In *Prdx1* K/O mice, NAC administration also recovered progesterone production and the levels of steroidogenic enzymes and CHOP (Fig. 6). In addition, the levels of serum progesterone and steroidogenic enzymes in *Prdx1* heterozygous mice showed the same pattern as in WT mice (Additional file 2: Figure S2). PRDX1 expression is upregulated following exposure to oxidative stress [10]. In agreement with this observation, we observed that Tm treatment increased PRDX1 expression, which was significantly recovered by NAC or TUDCA (Fig. 3c and d). We believe that the antioxidant enzyme PRDX1 is upregulated by the ER stress-associated oxidative environment of the mouse luteal phase. In the present study, NAC administration rescued luteal function by restoring progesterone production and reducing ER stress in the CL of *Prdx1* K/O mice (Fig. 6). Therefore, these results demonstrate that PRDX1 is a regulator of ER stress-induced ROS production that is required for maintaining progesterone production during the functional stage in mouse CL.

Several studies have demonstrated a crosstalk between the production of ROS and UPR signal-induced ER stress response [15, 37, 38]. Previously, we showed that UPR is involved in steroidogenic enzyme expression in hCG-stimulated Leydig cells [22] and regulation of CL function in mice luteal phase [18]. The UPR signaling pathways were activated in response to ER stress, which plays important roles in the regulation of CL function, and ER stress-mediated apoptosis occurs via three activated UPR pathways (PERK, ATF6, and IRE1) in the CL regression stage. To determine whether the loss in progesterone production depends on PRDX1, we attempted to identify a functional link between PRDX1 and UPR signaling (GRP78/BIP, p-eIF2α, p50ATF6, and p-IRE1) and ER-stress mediated apoptotic factors (p-JNK, CHOP, and cleaved caspase-3) in the functional stage using WT and *Prdx1* K/O mice. As shown in Fig. 5a and b, GRP78/BIP, p-eIF2α, p50ATF6, p-JNK, CHOP, and cleaved caspase-3 were upregulated in the CL functional stage in the *Prdx1* K/O mice. In addition, p50ATF6, p-eIF2α and cleaved caspase-3 were detected in the CL tissue of *Prdx1* K/O mice by immunohistochemical staining (Fig. 5c). Interestingly, although the exact molecular mechanism has not been fully elucidated, phosphorylation of the UPR marker IRE1 was not affected in the *Prdx1* K/O mice. Activation of GRP78/BIP and p50ATF6 were also significantly reduced by NAC administration in *Prdx1* K/O mice (Fig. 6c.). Therefore, the present study demonstrates a link between the antioxidant enzyme PRDX1 and progesterone production via regulation of UPR signaling and ER stress response. Consequently, PRDX1 deficiency activated the UPR signaling pathways and ER stress-mediated apoptosis in the CL functional stage. These novel observations suggest that PRDX1 may be component of a regulatory network for luteal functions that specifically reduce ROS.

## Conclusion

**Fig. 7:**
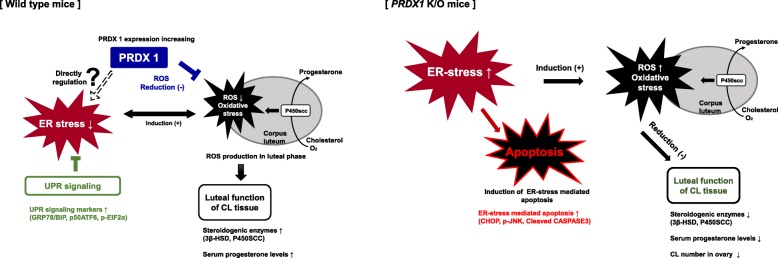
Roles of PRDX1 on progesterone production, and UPR signaling activation in CL tissue of mice. Graphical summary. *Right panel*; ROS reduction by PRDX1 during the luteal functional stage (48 h after PMSG/hCG injection) in WT mice decreased activated UPR signaling in response ER stress and increased steroidogenesis enzymes expression in the CL to promote progesterone production. *Left panel*; Increasing ROS by PRDX1 deficiency during the luteal functional stage of *Prdx1* K/O mice increased activated UPR signaling in response ER stress mediated apoptosis and decreased progesterone production related steroidogenesis enzymes expression in CL tissue and serum progesterone levels

In conclusion, our findings demonstrate that PRDX1 is an antioxidant enzyme, which is essential for preserving progesterone production against ER stress-associated oxidative stress in the CL functional stage (Fig. 7). PRDX1 deficiency decreased progesterone production via ER-stress induced ROS generation in CL tissue. Therefore, PRDX1 is required for the maintenance of luteal function via regulation of UPR signaling pathways in response to ER stress in mice. These findings highlight the basic mechanisms underlying PRDX1 antioxidant activity, which will enhance our understanding of the relationship between antioxidant enzymes and progesterone production in CL functional stage.

## Additional files

Additional file 1: **Figure S1.** Changes in PRDX family (2–6) protein levels in luteal phase of WT mice. Protein levels of PRDX 2–6 were confirmed in CL tissue by western blot analysis (A). The relative levels of PRDX proteins were obtained after normalization to β-actin levels. The histogram values of densitometry analysis were obtained using the Image J software. The bar graph data represent the least-squares means ± SEM of three independent experiments. *P < 0.05, **P < 0.01, and ***P < 0.001; Dunnett’s multiple comparison test compared to 16 h after PMSG/hCG injection. (DOCX 281 kb)

Additional file 2: **Figure S2.** Effects of PRDX1 on progesterone production in CL tissue using *Prdx1* heterozygous and K/O mice. We observed ovary morphology in wild type, *Prdx1* K/O and heterozygous mice (A). Serum progesterone levels were measured using a progesterone kit in *Prdx1* K/O and heterozygous mice (B). The levels of steroidogenic enzymes 3β-HSD and P450SSC were determined in the CL tissue using western blot analysis. The relative levels of the steroidogenic enzymes were normalized to β-actin levels (C). Western blotting analysis of the ER stress marker CHOP in CL tissue of *Prdx1* heterozygous and K/O mice. The relative levels of CHOP were normalized to β-tubulin levels (control). (D) The histogram values of densitometry analysis were obtained using the Image J software. The bar graphs represent the least-squares means ± SEM of three independent experiments. *P < 0.05, **P < 0.01, and ***P < 0.001; Dunnett’s multiple comparison test compared to 48 h after PMSG/hCG injection. (DOCX 331 kb)

## Abbreviations

3β-HSD: 3β-hydroxysteroid dehydrogenase
ANOVA: One-way analysis of variance
ATF6: Activating transcription factor-6
BSA: Bovine serum albumin
CAT: Catalase
CHOP: C/EBP homologous protein
CL: Corpus luteum
DAB: 3′-diaminobenzidine
ECL: Enhanced chemiluminescence
ER: Endoplasmic reticulum
GPX: Glutathione peroxidase
H_2_O_2_: Hydrogen peroxide
hCG: Human chorionic gonadotropin/mouse
HRP: Horse radish peroxidase
IHC: immunohistochemistry
IRE1 or ERN1: Inositol-requiring enzyme 1
JNK: cJUN NH2-terminal kinase
K/O: Knockout
NAC: N-acetylcysteine
NC: Nitrocellulose
P450scc: P450 cholesterol side-chain cleavage enzyme
PERK: Protein kinase RNA (PKR)-like ER kinase
PMSG: Pregnant mare’s serum gonadotropin
PRDX1: Peroxiredoxin 1
PRDXs: Peroxiredoxins
ROS: Reactive oxygen species
SDS-PAGE: Sodium dodecyl sulfate-polyacrylamide gel electrophoresis
SEM: Standard error of the mean
SOD: Superoxide dismutase
StAR: Steroidogenic acute regulatory
TBS: Tris-buffered saline
Tm: Tunicamycin
UPR: Unfolded protein response
WT: Wild type
