# Supplementary material for: Peroxiredoxin I maintains luteal function by regulating unfolded protein response
Source: Reprod Biol Endocrinol. 2018 Aug 15;16:79. doi: 10.1186/s12958-018-0396-0 (PMC6094449; doi:10.1186/s12958-018-0396-0)
Supplement: Supplementary file 1 — Figure S1. Changes in PRDX family (2–6) protein levels in luteal phase of WT mice. Protein levels of PRDX 2–6 were confirmed in CL tissue by western blot analysis (A). The relative levels of PRDX proteins were obtained after normalization to β-actin levels. The histogram values of densitometry analysis were obtained using the Image J software. The bar graph data represent the least-squares means ± SEM of three independent experiments. *P < 0.05, **P < 0.01, and ***P < 0.001; Dunnett’s multiple comparison test compared to 16 h after PMSG/hCG injection. (DOCX 281 kb) [file 12958_2018_396_MOESM1_ESM.docx]

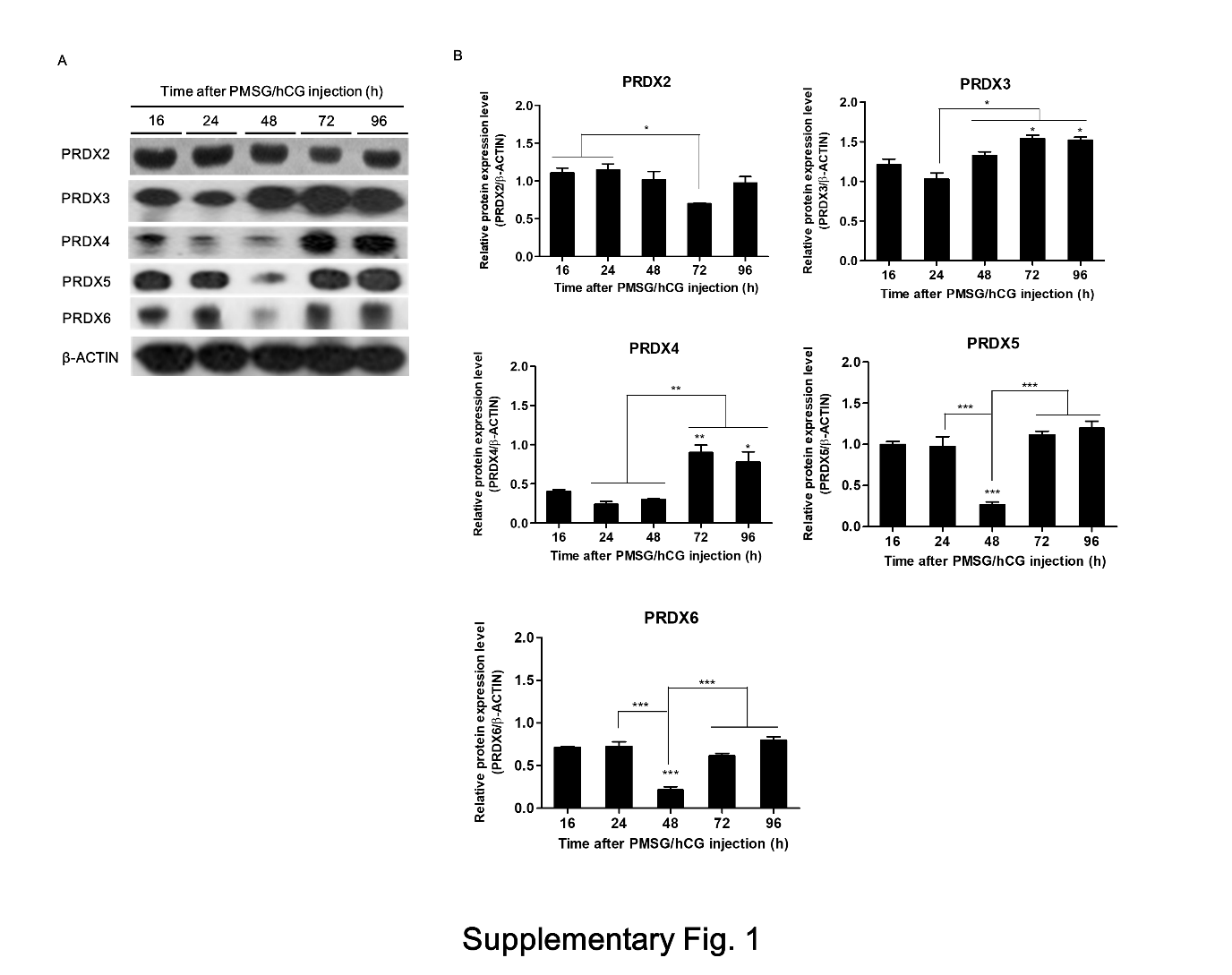


**Figure S1 Changes in PRDX family (2-6) protein levels in the luteal phase of CL tissue in wild type mice.** Protein levels of PRDX 2-6 were confirmed in CL tissue by western blot analysis (A). The relative levels of PRDX proteins were obtained after normalization to β-actin levels. The histogram values of densitometry analysis were obtained using the Image J software. The bar graph data represent the least-squares means ± SEM of three independent experiments. **P* < 0.05, ***P* < 0.01, and ****P* < 0.001; Dunnett's multiple comparison test compared to 16 h after PMSG/hCG injection.
