# Supplementary material for: Peroxiredoxin I maintains luteal function by regulating unfolded protein response
Source: Reprod Biol Endocrinol. 2018 Aug 15;16:79. doi: 10.1186/s12958-018-0396-0 (PMC6094449; doi:10.1186/s12958-018-0396-0)
Supplement: Supplementary file 2 — Figure S2. Effects of PRDX1 on progesterone production in CL tissue using Prdx1 heterozygous and K/O mice. We observed ovary morphology in wild type, Prdx1 K/O and heterozygous mice (A). Serum progesterone levels were measured using a progesterone kit in Prdx1 K/O and heterozygous mice (B). The levels of steroidogenic enzymes 3β-HSD and P450SSC were determined in the CL tissue using western blot analysis. The relative levels of the steroidogenic enzymes were normalized to β-actin levels (C). Western blotting analysis of the ER stress marker CHOP in CL tissue of Prdx1 heterozygous and K/O mice. The relative levels of CHOP were normalized to β-tubulin levels (control). (D) The histogram values of densitometry analysis were obtained using the Image J software. The bar graphs represent the least-squares means ± SEM of three independent experiments. *P < 0.05, **P < 0.01, and ***P < 0.001; Dunnett’s multiple comparison test compared to 48 h after PMSG/hCG injection. (DOCX 331 kb) [file 12958_2018_396_MOESM2_ESM.docx]

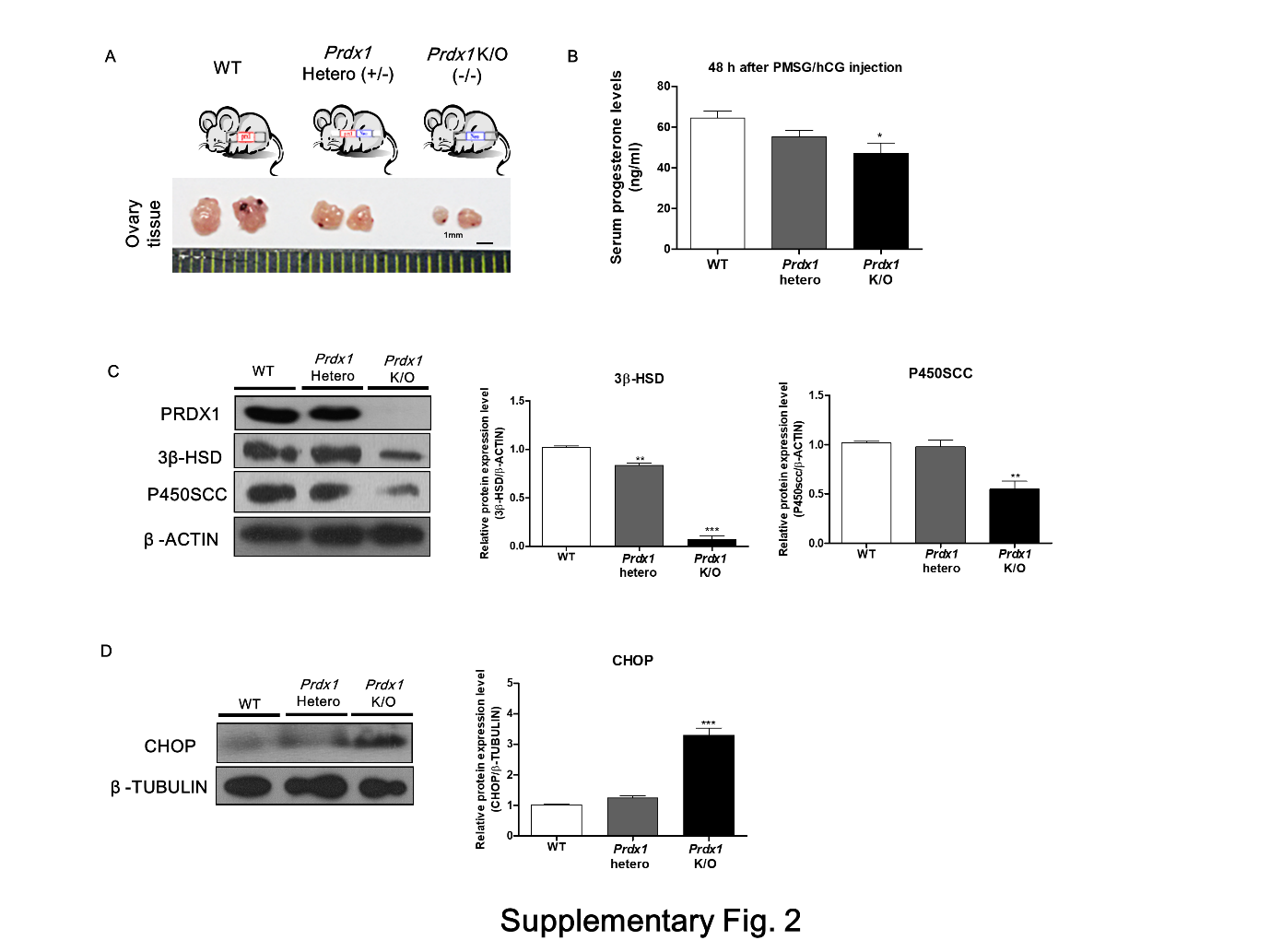


**Figure S2 Effects of PRDX1 on progesterone production in CL tissue using *Prdx1* heterozygous and K/O mice.** We observed ovary morphology in wild type, *Prdx1* K/O and heterozygous mice (A). Serum progesterone levels were measured using a progesterone kit in *Prdx1* K/O and heterozygous mice (B). The levels of steroidogenic enzymes 3β-HSD and P450SSC were determined in the CL tissue using western blot analysis. The relative levels of the steroidogenic enzymes were normalized to β-actin levels (C). Western blotting analysis of the ER stress marker CHOP in CL tissue of *Prdx1* heterozygous and K/O mice. The relative levels of CHOP were normalized to β-tubulin levels (control). (D) The histogram values of densitometry analysis were obtained using the Image J software. The bar graphs represent the least-squares means ± SEM of three independent experiments. **P* < 0.05, ***P* < 0.01, and ****P* < 0.001; Dunnett's multiple comparison test compared to 48 h after PMSG/hCG injection.
